# Employer-Paid Nonmedical Costs for Patients With Diabetes and End-Stage Renal Disease

**Published:** 2006-06-15

**Authors:** Sachin J Kamal-Bahl, Susan Pantely, Bruce Pyenson, Charles M Alexander

**Affiliations:** Outcomes Research and Management, Merck & Co, Inc; Milliman, Inc, New York, NY; Milliman, Inc, New York, NY; Outcomes Research and Management, Merck & Co, Inc, West Point, Pa

## Abstract

**Introduction:**

Disease conditions such as end-stage renal disease (ESRD), which have severe consequences of disability and mortality, can generate substantial costs for large employers providing life insurance and disability insurance benefits. This study is the first to examine such disease-related nonmedical costs for employers and models the following employer-paid costs for ESRD in patients with diabetes: 1) life insurance benefits, 2) disability benefits, and 3) cost of replacing a worker.

**Methods:**

We simulated a hypothetical cohort of 10,000 individuals with the age and sex distribution of a typical employee population in the United States. Data sources for the model parameters included the United States Renal Data System and proprietary life insurance and disability insurance claims databases. In addition, we used published information to identify the structures of typical employee benefits programs and annual salary information and to estimate the cost of replacing lost workers.

**Results:**

The study estimated that employers may incur life insurance costs of $55,055 per ESRD-related death, disability insurance costs of $31,671 per ESRD-related disability, and worker replacement costs of $27,869 per ESRD-related lost worker. Overall, the total monthly cost per employee with ESRD and diabetes was $5439.

**Conclusion:**

Our study finds that, other than the large direct medical costs documented in literature, ESRD onset also results in substantial nonmedical costs for employers. As employers continue to debate changes in the structure of future health plan benefits to reduce health care costs, they should consider potential indirect cost savings of providing affordable access to medical care that prevents or delays disability and mortality in their workers.

## Introduction

The prevalence of end-stage renal disease (ESRD) has continually increased over the past 2 decades (1). Between 1980 and 2001, prevalence increased fivefold from 271 to 1400 per million. Much of this growth can be attributed to an increase in the prevalence of diabetic nephropathy, the most common cause of ESRD. In addition to the clinical burden of ESRD, preventing or delaying its onset among patients with diabetes becomes especially crucial in reducing the economic impact of ESRD on the health care system.

Previous studies have reported substantial ESRD-related medical costs for health care payers such as Medicare, Medicaid, and employers (2,3). In one study, the change in costs from pre- to post-ESRD onset was nearly twice as high among those with diabetes as those without. (The adjusted change in mean costs from preonset to postonset was $57,973 for those with diabetes and $31,115 for those without diabetes [3].) Hence, recent research has focused on interventions to prevent or delay the onset of ESRD in patients with diabetes. These studies have projected substantial cost savings in individuals with type 2 diabetes and nephropathy from the perspective of third-party payers such as employers or health plans responsible for all direct medical costs (4). 

Employer-sponsored health insurance, which reaches more than three out of every five nonelderly Americans, plays an important role in providing affordable access to preventive health care and medical interventions that may prevent or delay the onset of conditions such as ESRD in patients with diabetes (5). However, increases in health care costs for employers over the past decade have resulted in many employers scaling back employee health insurance benefits and some eliminating health coverage (6). Even as employers have cut back on health insurance benefits, most have continued to provide other benefits such as life insurance and disability insurance to their employees (7). Whereas 62% of all workers received health coverage from their employer in 2003, 76% had term life insurance, and 67% had disability insurance in the same year (7,8). Similarly, although only one in six (16%) workers who received medical coverage from their employer did not have to pay for some or all of it, at least one third of those who received coverage through their employer had their company pay the entire cost of their disability (33%), basic term life insurance (38%), and accidental death and dismemberment insurance (35%) (7). When a worker dies or becomes disabled, these benefits provide cash payments to the survivors or to the disabled worker. Because large employers often effectively self-insure or purchase experience-rated programs for life insurance and disability insurance benefits, disease conditions that have severe consequences of disability and mortality can generate substantial costs for employers providing such benefits.

Although numerous studies have documented employers' direct medical costs for workers' disease conditions (1-3,9), we are not aware of any studies that have estimated the economic burden from nonmedical costs, such as life insurance and disability insurance payments connected to a medical condition. Because ESRD is an important cause of mortality and disability in the working-age population, it may also be associated with substantial nonmedical costs for employers in particular. Delaying (or avoiding) the onset of ESRD can consequently affect the cost of life insurance and disability benefits. It can also affect employer costs when there is a need to replace a worker. However, there appear to be no estimates of such costs available in the literature. To better understand the magnitude of the overall cost implications of ESRD in patients with diabetes, a study was undertaken to identify and measure the nonmedical costs for employers after ESRD onset. This study provides actuarial estimates of three types of employer-paid costs for ESRD in patients with diabetes: 1) employer-paid life insurance benefits, 2) employer-paid disability benefits, and 3) the cost of replacing a worker. The costs are estimated for typical large employers that provide comprehensive benefits to their employees and have self-insured or experience-rated programs, such as those in the automobile industry.

## Methods

### Model population 

The study simulated a hypothetical cohort of 10,000 male and female employees aged 20 years and older. We used standard demographics available from the 2004 Milliman Health Cost Guidelines to model the age and sex distribution of a typical employee population in this cohort (10). The demographics in these guidelines, developed by Milliman, Inc, one of the largest actuarial firms in the United States, have been adjusted from U.S. Department of Labor demographics based on data from large insurers and are more reflective of an insured population.

### Data sources 

The study used several data sources to model ESRD-related nonmedical costs. These data sources include databases produced by the federal ESRD program and proprietary life insurance and disability insurance claims databases. In addition, we used published information to identify the structures of typical employee benefits programs and annual salary information and to estimate the cost of replacing employees lost to long-term disability or death.

### Model calculations and estimates 

Table 1 summarizes the model inputs and data sources used in the calculation of each type of nonmedical cost.

**Life insurance costs**

Life insurance provided by employers is structured as group insurance, which means the policyholder for insured benefits is the employer, not the employee. Group life insurance benefits are typically set as a multiple of salary — typically one to three times annual salary. Large employers typically finance group life insurance through effective self-insurance or experience-rated programs. This means that large employers bear the direct costs: if life insurance costs are higher or lower than expected, the employer bears the extra cost or has the benefit of lower-than-expected costs.

To estimate life insurance costs, age- and sex-specific prevalence rates of ESRD and diabetes were determined from the 2003 United States Renal Data System (USRDS) (11). The USRDS is a national database characterizing the ESRD population and providing estimates of prevalence and incidence of ESRD with trends in annual mortality rates. To produce ESRD-related mortality rates with greater precision for the age distribution of our hypothetical cohort, the mortality rates from the USRDS were interpolated using the slopes in the 2001 Milliman Basic Mortality Tables (12) and insurance industry standard methodology (22). The typical benefits of life insurance provided by a large employer were then estimated. The components that were defined included 1) group life insurance coverage expressed as a multiple of salary and 2) salary by age and sex. We reviewed published life insurance and benefits trade industry references to identify a common benefit plan offered by large employers for group life insurance. According to these sources, 100% of salary is the most common plan design and is used in the base-case analysis (13-15). To test the sensitivity of the model, we also modeled other common benefits of 150% and 200% of salary in our analysis. We used U.S. Department of Labor sources to estimate annual salary by age and sex in large-employer firms (16,17). In the sensitivity analysis, we varied the salary by 25% higher and 25% lower. Finally, the life insurance cost to the employer for employees with diabetes and ESRD was calculated as the sum product of the prevalence rates of ERSD and diabetes, the mortality rates, and the benefit amount.

**Disability insurance costs**

Disability benefits provided by employers follow a similar financing and regulatory structure as life insurance benefits. Depending on the benefit design, *disability* is defined as the inability to work in any occupation or in one's own occupation. Group disability coverage replaces a portion of the income that was earned by a disabled worker before the disability. Employers provide two distinct types of disability insurance to employees (23,24). These include 1) short-term disability, which provides cash payments beginning when sick days or paid time off are exhausted and continues until long-term disability begins; and 2) long-term disability, which provides cash payments after a 3- to 9-month elimination period following the date of disability. The most common income replacement payment across short-term disability and long-term disability insurance plans is 60% to 80% of final salary. Disability benefits are paid monthly while the person remains both alive and disabled. Group long-term disability benefits are typically subject to a maximum duration until age 65 or death. However, insurers do sell products that continue payments beyond age 65 until death or for some specified period, such as 5 years. Most plans reduce their payment by the amount of any other disability benefits provided through other sources, such as Social Security Disability Insurance, worker's compensation, pension benefits, and disability plans with other employers. Most group long-term disability programs do not contain inflation adjustment benefits.

Most employers who provide disability plans offer both long-term disability and short-term disability plans. Long-term disability benefits are commonly designed to begin when short-term disability benefits expire so that benefits continue without interruption for disabled employees. Given the chronic nature of ESRD, most workers with the condition are expected to transition from short-term disability to long-term disability unless they receive a kidney transplant. Hence, we modeled short-term disability and long-term disability benefit costs together based on the most common plan design features identified from published references (Table 2) (18,19).

The first step was to estimate the rate of ESRD-related disability claims. This was done by assuming that 90% of all new cases of ESRD due to diabetes (stratified by age and sex) identified from the USRDS would become eligible for disability benefits within the calendar year. This assumption was based on our discussions with three insurance company medical directors who specialize in disability insurance. Because the assumption of 90% was not based on published data, we have tested this assumption as part of our sensitivity analysis.

Next, we estimated the present value of all expected future disability benefits (PVFB) that will be made to claimants who become disabled due to ESRD and diabetes in that year. This is because, unlike life insurance benefits, which are one-time lump-sum benefits, disability benefits may be paid for many years after an individual initially becomes eligible for benefits. For an individual with a disability, the PVFB as of the end of the elimination period (i.e., period after which disability payments begin) may be expressed as a function of the monthly benefit amount, continuance factors, and the discount rate.

**Benefit amount**

We assumed that the monthly benefit amount was equal to 60% of the employee's monthly income, reduced by 45% for the impact of benefit offsets from other sources. An employee's monthly income was calculated from the same age- and sex-specific annual salary assumptions used to model life insurance costs in the previous section. The 60% income replacement ratio represents the most typical plan design (Table 2), and the 45% reduction for benefit offsets represents the approximate difference in cost between disability plans that do not contain offsets (which are rare) and those that do contain offsets (which are the vast majority of plans). The 45% reduction estimate was obtained through an analysis of the rating manuals used by three group disability insurers for new business sold in 2004. As part of the sensitivity analyses, we assessed the impact of assuming either a 25% or a 75% reduction in the benefit amount.

**Continuance factors**

Continuance factors represent the probability that an individual with a disability is eligible for benefits in month *t*. Continuance decreases as the value of *t* (i.e., the length of the claim) increases, because disability benefits may end as the result of death or recovery. These factors were developed through analysis of Milliman's proprietary database containing disability claims pooled from four large disability insurers. It contains information for approximately 26,300 claims, covering a period from approximately 1998 to 2001. We developed continuance factors for ESRD-related disabilities by studying the patterns of claim termination (i.e., deaths and recoveries) for the disability claims contained in this database with *International Classification of Diseases, Ninth Revision, Clinical Modification* (*ICD-9-CM*) codes for renal failure.

**Discount rate**

The discount rate used to compute the PVFB reflects the anticipated returns on the type of investments commonly purchased by disability insurers to fund the future benefit payments. Most disability insurers invest in investment-grade bonds and mortgages. As of mid-December 2004, new-money rates for these types of investments were approximately 5.5%, which we used to compute the PVFB. We also conducted sensitivity testing using interest rates of 4.5% and 6.5%. Finally, the disability insurance cost to the employer for employees with diabetes and ESRD was calculated as the sum product of the disability claim rates and the PVFB.

**Cost of replacing a worker**

We made two assumptions in estimating the costs that employers have when replacing a lost worker. First, because recoveries from renal failure are uncommon, we assumed that employees who could have returned to their jobs because of improvements in health (e.g., after renal transplant) would have been replaced. Second, we did not include ESRD-related deaths in our estimate of number of employees that would need to be replaced. Instead, we assumed that all employees with diabetes and ESRD who died would be receiving disability benefits before death, and hence, ESRD-related disability rates account for all employees who will have to be replaced. We used the friction cost approach and published estimates for estimating the employer's cost of replacing a lost worker (20,21). For our base-case analysis, we assumed that the expected cost of replacing a lost worker who becomes disabled because of diabetes and ESRD is 50% of the worker's annual salary. We used age- and sex-specific annual salary assumptions consistent with those in the life insurance section and disability rates obtained in the disability insurance section. We conducted sensitivity analysis using factors of 25% and 100% of employee salary to project the cost of replacing a worker.

## Results

### Base-case results 

Table 3 summarizes the estimates of the ESRD-related nonmedical costs paid by employers for employees with diabetes under the base-case scenario. The life insurance cost was estimated to be $742 per employee with diabetes and ESRD per month. The estimates for total disability insurance cost and the cost of replacing a worker were threefold higher at $2375 per employee with diabetes and $2322 per employee with ESRD per month. Overall, the total monthly cost per employee with ESRD and diabetes was $5439. The average life-insurance cost per ESRD-related death was $55,055. The average cost per employee with an ESRD-related disability was $31,671, whereas the average cost of replacing an employee with diabetes and ESRD was estimated to be $27,869.

### Sensitivity analysis results 

Table 4 presents results from one-way sensitivity analyses.

**ESRD-related life insurance costs**

The base-case analysis estimated the cost of life insurance benefits for a life insurance benefit of 1 times the annual salary. Life insurance benefits of 1.5 and 2 times the salary are also common benefits among large-employers. Table 4 shows that the estimates for life insurance costs are directly proportional to the amount of benefit (i.e., multiple of annual salary). With a life insurance benefit of 2 times the salary, the monthly cost per employee with ESRD and diabetes doubles to $1484 and the average cost per ESRD–diabetes-related death to $110,110. Similarly, when salary estimates were modeled at 25% above and 25% below the salary estimates used in the base-case analysis, the cost to employer was directly proportional to the increase and decrease in the annual salary assumption, respectively.

**ESRD-related disability insurance costs**

Sensitivity analysis results using the main parameter assumptions for estimating disability insurance costs are shown in Table 4. When the baseline assumption of the proportion of employees with ESRD due to diabetes who qualify for disability benefits was decreased from 90% to 75%, the monthly cost per employee with ESRD and diabetes decreased proportionally. However, as expected, the cost per worker with an ESRD-related disability remained the same. On the other hand, when the percentage reduction in disability benefits due to offsets for benefits from other sources was varied from 45% in the base case to 25% and 75%, the results were inversely proportional to the change. Finally, sensitivity analysis using interest rates of 4.5% and 6.5% changed the results very slightly.

**ESRD-related costs of replacing a worker**

Because estimates for the cost of replacing a worker vary greatly, we projected the cost of replacing a worker assuming replacement costs are 25% and 100% of a worker's annual salary. Table 4 shows that the sensitivity analysis results are directly proportional to the multiple of salary used, and the replacement cost per worker lost due to ESRD and diabetes ranges from $13,935 to $55,738.

## Discussion

In the literature, ESRD has been associated with enormous direct medical costs among patients with diabetes. This study finds that ESRD among workers with diabetes is also associated with substantial nonmedical costs for employers. For instance, one study has reported that ESRD onset in patients with diabetes increased annual direct medical costs by $57,973 per patient. Our study finds that ESRD onset also results in monthly nonmedical costs of $5439 per employee with diabetes and ESRD. Under base-case assumptions, the study estimates that employers may incur life insurance costs of $55,055 per ESRD-related death, disability insurance costs of $31,671 per ESRD-related disability, and worker replacement costs of $27,869 per ESRD-related lost worker. Hence, reducing ESRD-related disability and mortality can translate into savings for large employers who self-insure or purchase experience-rated programs.

The study findings should be viewed in light of the following limitations. First, the results are based on various model assumptions such as worker salary, mortality, and disability rates. Actual experience for an employer or insurer will vary from the results shown here based on its employee population's demographics, health status, salaries, benefit designs, technological advances, and other factors. Second, we did not calculate employer costs due to worker absenteeism or presenteeism at the beginning of the illness before the worker transitions onto disability benefits and needs to be replaced. Hence, our estimates on the cost for replacing a lost worker represent the lower bound of all productivity costs faced by employers due to ESRD onset in workers with diabetes. Finally, the results apply only to large employers who self-insure or purchase experience-rated programs for life insurance and disability insurance benefits.

Over the past few years, employers have been under intense financial pressure because of large increases in health care costs. Consequently, many have shifted some of this cost onto their employees in the form of increased premium contributions, deductibles, and copayments (7). To curb growth in prescription drug expenditures, many employers have increasingly moved to tiered formulary structures and higher drug copayment requirements. Others have completely eliminated health coverage, prescription drug coverage, or both. Studies have shown that increased cost-sharing results in lower medical care and prescription use among patients and substantial cost savings for health plans and employers (25-30). Hence, although medical therapies that prevent or delay the onset of ESRD in patients with diabetes are available, their use may be limited among workers with diabetes because of lack of coverage or higher cost sharing. For instance, certain medications shown to reduce ESRD in patients with diabetes have been reported to be frequently placed on the highest copayment tier of the formulary (31). Studies have shown that the likelihood of use of such medications is significantly lower among patients in tiered copayment drug plans (25,26,28). Whether such strategies by large employers to reduce costs of employee health benefits result in increased outlays for other employee benefits requires further empirical study. However, it appears that if scaling back health benefits to reduce health care costs were to adversely affect workers' access to essential prescription and medical services, and consequently their health, then employers may have increased costs for life insurance, disability insurance, and employee turnover.

This study is the first to examine such disease-related nonmedical costs for employers and focuses on ESRD in people with diabetes. ESRD is an example of a disease condition that can be prevented or delayed with appropriate medical interventions, but the disease results in high rates of disability and mortality. Although the cost estimates in this study represent employers' nonmedical costs for ESRD in employees with diabetes, nonmedical costs for employers may be enormous for other disease conditions such as heart disease, cancer, and lung diseases, which have a substantially higher prevalence and are leading causes of mortality and morbidity. As employers continue to debate changes in the structure of future health benefits, they should consider the potential for indirect cost savings by providing affordable access to medical care that prevents or delays disease-related disability and mortality in their employees.

## Figures and Tables

**Table 1:** Model Inputs and Sources Used to Calculate Employer-Paid Nonmedical Costs of Employees with Diabetes and End-Stage Renal Disease (ESRD), by Type of Cost

**Model Input^a^**	**Data Source**
**Life insurance costs**	
% ESRD-related deaths	
% with diabetes and ESRD	USRDS (11)
% ESRD-related deaths	USRDS (11) and Milliman Basic Mortality Tables (12)
Life-insurance benefit amounts	
Typical benefit as % of salary*	Published references (13-15)
Employee salary	U.S. Department of Labor (16,17)
**Disability insurance costs**	
% ESRD-related disability claims	
% with diabetes and ESRD	USRDS (11)
% with ESRD-related disability*	Assumption based on medical director interviews
Disability benefit amounts	
Typical benefit as % of salary*	Published references (18,19)
Length of disability claim (i.e., continuance)	Milliman, Inc proprietary disability database
Discount rate	Assumption based on expected returns on typical investments by disability insurers
Employee salary	U.S. Department of Labor (16,17)
**Costs of replacing a worker**	
% workers lost due to ESRD	
% with diabetes and ESRD	USRDS (11)
% with ESRD-related disability	Assumption based on medical director interviews
Employee turnover amount	
Turnover costs as % of salary*	Published references (20,21)
Employee salary	U.S. Department of Labor (16,17)

USRDS indicates United States Renal Data System

a All model inputs except those marked with an asterisk (*) were age and sex specific.

**Table 2:** Most Common Employer-Provided Disability Plan Provisions

**Provision**	**Short-term Disability**	**Long-term Disability**
Benefit as % of salary	60	60
Maximum monthly benefit	$4000	$6000
Elimination period	7 days	180 days
Maximum benefit period	26 weeks	To age 65
Definition of disability	Unable to work in own occupation	Unable to work in own occupation for 24 months or in any occupation thereafter
Social Security offsets	Primary and family benefits	Primary and family benefits

**Table 3:** Base-Case Results for Employer-Paid ESRD-related Nonmedical Costs for Employees With Diabetes

**Type of Cost**	**Life Insurance, $**	**Disability Insurance, $**	**Replacement, $**	**Total, $**
Monthly cost per employee with ESRD and diabetes	742	2,375	2,322	5,439
Cost per ESRD–diabetes-related death	55,055	NA	NA	55,055
Cost per worker disabled due to ESRD-diabetes	NA	31,671	NA	31,671
Cost per worker lost due to ESRD–diabetes-related disability or death	NA	NA	27,869	27,869

ESRD indicates end-stage renal disease; NA, not applicable.

**Table 4:** One-way Sensitivity Analysis Results for Employer-Paid ESRD-related Nonmedical Costs for Employees With Diabetes

**Sensitivity Test**	**Monthly Cost per Employee With ESRD and Diabetes, $**	**Average Cost per ESRD–Diabetes-related Death or Worker With Disability, $**
**Life insurance costs**		
Base case^a^	742	55,055
Benefit = 1.5 x Annual salary	1,113	82,583
Benefit = 2.0 x Annual salary	1,484	110,110
Salary = .75 x Base-case salary	594	44,044
Salary = 1.25 x Base-case salary	928	68,819
**Disability insurance costs**		
Base case^b^	2,375	31,671
Proportion of ESRD–diabetes patients qualifying for disability benefits = 75%	1,979	31,671
Reduction in benefit amount for other benefits such as Social Security Disability Insurance = 25%	3,239	43,188
Reduction in benefit amount for other benefits such as Social Security Disability Income = 75%	1,080	14,396
Discount rate = 6.5%	2,291	30,544
Discount rate = 4.5%	2,468	32,902
**Costs of replacing a lost worker**		
Base case^c^	2,322	27,869
Cost to replace lost worker = 25% of annual salary	1,161	13,935
Cost to replace lost worker = 100% of annual salary	4,645	55,738

a ESRD indicates end-stage renal disease.

b Assumes 90% of ESRD–diabetes cases are eligible for disability benefits; 45% reduction in disability benefit amount for other benefits, such as Social Security Disability Insurance; and discount rate of 5.5%.

c Assumes cost to replace lost worker = 50% of annual salary.
